# Physiological Index, Weight Control Behavior, and Quality of Life Related to Obesity-related Metabolic Syndrome in Korean Adults

**Published:** 2017-03

**Authors:** Jeong Won HAN

**Affiliations:** College of Nursing, Kosin University, Amnam-dong, Seo-gu, Busan, Korea

## Dear Editor-in-Chief

According to WHO, 53.5–69.5% of adult men and 47.2–61.8% of adult women were classified as overweight in seven European countries. Among adults classified as overweight, 7.7–26.0% of men and 9.0–21.0% of women were classified as obese (1). BMI is not always related to the degree of insulin resistance, although BMI is an important indicator for diagnosing obesity. Furthermore, a low occurrence of diseases is associated with obesity, although they had the same degree of obesity. They were named metabolically healthy obese adults (2, 3). The adults with metabolic syndrome have a 2.4 and 6.0 times higher risk of a heart attack, for men and women, respectively, than adults without metabolic syndrome. Moreover, the mortality rate due to cardiovascular disease increases more than 5 times for adults with metabolic syndrome. Therefore, having a metabolic syndrome oriented approach is important for managing obese adults who are at high-risk for cardiovascular disease (4, 5).

This study included 19,853 people who participated in the 5^th^ and 6^th^ Korean National Health and Nutrition Examination Survey.

Analyses showing indices with an area under the ROC curve were investigated by verifying the ROC curve for indices with high sensitivity and specificity by group using physiological indices (excluding the 5 criteria of NCEP-ATP III) showing differences among groups. For men, HbA1C was a sensitive index for non-obese identified as sensitive indices for obese healthy men. ALT and HbA1C were sensitive indices for obese unhealthy men. For women, HbA1C was a sensitive index for non obese unhealthy adults, while ALT and LDL-cholesterol were identified as sensitive indices for obese healthy women. ALT and HbA1C were sensitive indices for obese unhealthy women.

Non-obese healthy adults used 1.67 times more food uptake reduction methods and were 2.70 times more likely to skip a meal than non-obese unhealthy adults. Obese healthy adults were more likely to engage in flexibility exercises (1.05), use food intake reduction (1.21), take weight-loss pills without a doctor’s prescription (2.62), and take prescribed weight-loss pills (1.43) than non-obese healthy adults. Obese unhealthy adults were more likely to engage in strenuous physical exercise (1.02) and take prescribed weight-loss pills 1.45 times more than non-obese healthy adults. However, non-obese healthy adults engaged in walking exercise 1.03 times more than obese unhealthy adults. When data from men were adjusted for age and income, obese healthy adults exercised 1.54 times more and had 1.60 times more daily activity than non-obese healthy adults. However, self-management was 1.82 times higher among non-obese healthy adults than among obese unhealthy adults. When data from women were adjusted for age and income, obese healthy adults exercised 1.50 times more and had 1.59 times more daily activity than non-obese healthy adults. However, anxiety/depression was 1.20 times higher among unhealthy adults than among non-obese healthy adults (Table 1 and 2).

**Table 1:** Weight control activities by groups

**Variable** **Exercise**	**Non-obese unhealthy OR(95% CI)**	**Obese-healthy OR(95% CI)**	**Obese-unhealthy OR(95% CI)**
Strenuous physical activity	1.01(0.98–1.03)	1.02(0.99–1.04)	1.02(1.00–1.05)*
Moderate physical activity	1.02(0.99–1.04)	1.00(0.98–1.02)	1.01(0.99–1.03)
Walking	0.98(0.96–1.00)	0.98(0.97–1.00)	0.97(0.96–0.99)*
Muscle strength exercise	0.97(0.91–1.03)	0.99(0.96–1.04)	0.98(0.94–1.02)
Flexibility exercises	1.01(0.96–1.01)	1.05(1.02–1.08)*	0.98(0.94–1.01)
Fasting	0.40(0.12–1.33)	1.03(0.68–1.56)	1.08(0.69–1.69)
Reduce amount of food	0.60(0.49–0.74)*	1.21(1.07–1.37)*	0.91(0.79–1.04)
Go skip a meal	0.37(0.21–0.64)*	0.92(0.75–1.14)	0.87(0.67–1.13)
Take weight-loss pills without a doctor's prescription	1.01(0.26–3.88)	2.62(1.43–4.79)*	1.45(0.67–3.12)
Take prescribed weight-loss pills	0.44(0.15–1.31)	1.43(1.03–1.99)^*^	1.45(1.00–2.09)^*^
Take oriental medicine	0.36(0.13–1.05)	1.20(0.82–1.76)	1.27(0.81–1.98)
Take health functional food	1.22(0.79–1.87)	1.03(0.79–1.32)	0.77(0.57–1.04)
One food diet	0.62(0.22–1.77)	1.03(0.70–1.51)	0.74(0.45–1.24)

Reference group=non-obese healthy; OR, odds ratio; CI, confidence interval;

* *p*<0.05

**Table 2:** The quality of life comparisons by groups

**Variables**		**Male**			**Female**		
		**Unadjusted**	**Age adjusted**	**Age+income adjusted**	**Unadjusted**	**Age adjusted**	**Age+income adjusted**
		**OR(95% CI)**	**OR(95% CI)**	**OR(95% CI)**	**OR(95% CI)**	**OR(95% CI)**	**OR(95% CI)**
Non-obese unhealthy	Exercise capacity	0.49(0.34–0.71)	0.89(0.61–1.29)	0.86(0.59–1.25)	0.39(0.29–0.52)	1.00(0.75–1.35)	1.03(0.77–1.37)
	Self-management	1.13(0.68–1.89)	1.31(0.79–2.19)	1.36(0.81–2.29)	.091(0.61–1.35)	1.33(0.89–1.98)	1.32(0.88–1.99)
	Daily activity	1.05(0.68–1.62)	1.14(0.75–1.75)	1.16(0.76–1.79)	0.78(0.55–1.09)	0.99(0.71–1.39)	1.03(0.74–1.45)
	Pain discomfort	0.85(0.63–1.15)	0.90(0.67–1.22)	0.91(0.67–1.22)	0.83(0.66–1.05)	0.92(0.76–1.19)	0.94(0.75–1.18)
	Anxiety and depression	0.75(0.52–1.08)	0.77(0.53–1.12)	0.75(0.51–1.09)	1.07(0.81–1.42)	0.96(0.72–1.26)	0.95(0.72–1.25)
Obese-healthy	Exercise capacity	1.36(0.99–1.86)	1.53(1.10–2.11)*	1.54(1.11–2.14)*	1.92(1.61–2.29)*	1.51(1.25–1.82)*	1.50(1.25–1.81)*
	Self-management	1.37(0.88–2.11)	1.30(0.83–2.21)	1.31(0.84–2.03)	0.92(0.65–1.31)	1.05(0.74–1.49)	1.06(0.74–1.50)
	Daily activity	1.64(1.08–2.50)*	1.62(1.05–2.49)*	1.60(1.04–2.47)*	1.13(0.89–1.42)	1.20(0.95–1.52)	1.19(0.94–1.51)
	Pain discomfort	0.97(0.79–1.18)	0.96(0.78–1.17)	0.96(0.78–1.17)	0.84(0.73–0.95)	0.87(0.76–0.99)	0.88(0.77–1.01)
	Anxiety and depression	1.15(0.86–1.54)	1.14(0.85–1.53)	1.13(0.84–1.51)	0.99(0.85–1.17)	0.98(0.83–1.15)	0.98(0.83–1.15)
Obese-unhealthy	Exercise capacity	0.71(0.53–0.95)	0.85(0.63–1.16)	0.82(0.61–1.10)	0.35(0.29–0.43)*	0.64(0.51–0.79)*	0.63(0.51–0.79)*
	Self-management	0.63(0.39–1.01)*	0.59(0.37–0.96)*	0.55(0.35–0.55)*	1.05(0.88–1.25)	0.98(0.82–1.18)	0.98(0.82–1.17)
	Daily activity	1.03(0.72–1.47)	1.06(0.74–1.52)	1.10(0.77–1.59)	0.89(0.71–1.14)	1.04(0.82–1.33)	1.05(0.82–1.34)
	Pain discomfort	0.94(0.74–1.19)	0.96(0.76–1.21)	0.97(0.76–1.23)	0.88(0.76–1.03)	0.97(0.83–1.13)	0.98(0.84–1.15)
	Anxiety and depression	1.10(0.83–1.47)	1.12(0.84–1.49)	1.08(0.81–1.44)	0.83(0.61–1.13)	1.17(0.97–1.49)	1.20(1.01–1.54)*

Reference group=non-obese healthy; M, mean; OR, odds ratio; CI, confidence interval;

* *P*<0.05
